# Subtypes of Alzheimer's Disease Display Distinct Network Abnormalities Extending Beyond Their Pattern of Brain Atrophy

**DOI:** 10.3389/fneur.2019.00524

**Published:** 2019-05-28

**Authors:** Daniel Ferreira, Joana B. Pereira, Giovanni Volpe, Eric Westman

**Affiliations:** ^1^Division of Clinical Geriatrics, Centre for Alzheimer Research, Department of Neurobiology, Care Sciences, and Society, Karolinska Institutet, Stockholm, Sweden; ^2^Department of Physics, University of Gothenburg, Gothenburg, Sweden; ^3^Department of Neuroimaging, Centre for Neuroimaging Sciences, Institute of Psychiatry, Psychology, and Neuroscience, King's College London, London, United Kingdom

**Keywords:** Alzheimer's disease, graph theory, neurofibrillary tangles, structural MRI, subtypes, heterogeneity

## Abstract

Different subtypes of Alzheimer's disease (AD) with characteristic distributions of neurofibrillary tangles and corresponding brain atrophy patterns have been identified using structural magnetic resonance imaging (MRI). However, the underlying biological mechanisms that determine this differential expression of neurofibrillary tangles are still unknown. Here, we applied graph theoretical analysis to structural MRI data to test the hypothesis that differential network disruption is at the basis of the emergence of these AD subtypes. We studied a total of 175 AD patients and 81 controls. Subtyping was done using the Scheltens' scale for medial temporal lobe atrophy, the Koedam's scale for posterior atrophy, and the Pasquier's global cortical atrophy scale for frontal atrophy. A total of 89 AD patients showed a brain atrophy pattern consistent with typical AD; 30 patients showed a limbic-predominant pattern; 29 patients showed a hippocampal-sparing pattern; and 27 showed minimal atrophy. We built brain structural networks from 68 cortical regions and 14 subcortical gray matter structures for each AD subtype and for the controls, and we compared between-group measures of integration, segregation, and modular organization. At the global level, modularity was increased and differential modular reorganization was detected in the four subtypes. We also found a decrease of transitivity in the typical and hippocampal-sparing subtypes, as well as an increase of average local efficiency in the minimal atrophy and hippocampal-sparing subtypes. We conclude that the AD subtypes have a distinct signature of network disruption associated with their atrophy patterns and further extending to other brain regions, presumably reflecting the differential spread of neurofibrillary tangles. We discuss the hypothetical emergence of these subtypes and possible clinical implications.

## Introduction

Three subtypes of Alzheimer's disease (AD) based on the spread of neurofibrillary tangles (NFT) have recently been identified (1): typical AD has rather balanced NFT counts in the hippocampus and the association cortex; limbic-predominant AD has NFT counts predominantly in the hippocampus; and hippocampal-sparing AD has NFT counts predominantly in the association cortex. Structural magnetic resonance imaging (sMRI) can reliably track these subtypes *in vivo* (2). Indeed, the subtypes have been successfully identified by using both advanced methods for MRI data analysis (3–11) and clinically in-place visual rating scales of brain atrophy (12–16). A fourth subtype with no or minimal signs of gray matter atrophy (i.e., minimal atrophy AD), but comparable clinical severity, has also been described (6, 7, 10, 12–16).

However, it is still unknown how and why these patterns of NFT and brain atrophy do emerge. The “staging hypothesis” stands on the Braak and Braak (17) staging scheme, assuming a stereotypical pattern of spread with the NFT initiating in the entorhinal cortex and then progressively occupying the association cortex. According to this theory, minimal atrophy AD would be the earliest presentation, progressing to limbic-predominant AD, and finally to typical AD. However, the discovery of the hippocampal-sparing subtype challenges this theory, since NFT can be found in the association cortex while the medial temporal lobe is largely spared (1). Alternatively, the “distinct subtypes hypothesis” conciliates these contradictory findings by recognizing actual heterogeneity in disease expression (1, 8, 12). To note, there is growing evidence supporting the idea of distinct subtypes by proving, for instance, different patterns of atrophy leading the AD patients to the same clinical stage (1, 8, 11, 12, 15).

Although the “distinct subtypes hypothesis” is certainly attractive, little is known about the factors that drive the NFT to be expressed so differently across these subtypes. Two recent studies have shown that factors such as cognitive reserve and cerebrovascular pathology may play a role (13, 15). Findings showing different APOE ε4 distribution, age of onset, and CSF biomarker profiles across these subtypes have also shed some light into this issue (1–4, 6, 8, 9, 12, 15, 18). However, the underlying biological mechanisms that determine this differential NFT expression are still completely unknown. The finding of misfolded tau proteins first developing intraneuronally and then spreading from neuron to neuron through brain networks opens a promising door (19–21). In particular, sMRI and the study of brain networks can reveal the underlying pathology and its spread (2, 4, 11, 22, 23), thus, possibly helping to understand the emergence of these subtypes through different brain networks.

Brain networks can be investigated using concepts from graph theory (24). Within this framework, the brain is modeled as a network (the human connectome) (25), which is represented as a set of nodes connected by edges. In sMRI data, the nodes correspond to anatomical brain regions and the edges to the association between those regions, as estimated by statistical correlations (26). A connectivity matrix (brain graph) is thus created from all the possible pairwise correlations, and network properties can be investigated through several graph measures. For instance, global efficiency is a measure of integration, that is, the capacity of the brain to rapidly combine information from distributed brain regions (27). Transitivity, modularity, and local efficiency are measures of segregation, that is, the biologically meaningful feature of the brain to enable highly specialized processing through densely interconnected communities of regions (28–30). Graph theory provides rich information on the networks beyond the regional pattern of brain atrophy (31). Applied to the subtypes, graph theory is expected to provide critical insights on how network disruption contributes to cognitive impairment, for instance, in subtypes such as hippocampal-sparing or minimal atrophy AD that lack atrophy in the medial temporal lobe but show memory impairment comparable to typical and limbic-predominant AD (11, 12).

The aim of the current study was to investigate potential differences in network topology underlying the AD subtypes. We hypothesized that the typical, limbic-predominant, and hippocampal-sparing subtypes of AD would show regional changes in network topology mostly paralleling the regional pattern of atrophy that defines each subtype, but also extending to other brain regions reflecting the involved networks (31). Because previous studies have shown that network disruption occurs before overt brain atrophy (31, 32), we also hypothesized that minimal atrophy AD would show changes in network topology in the absence of overt brain atrophy. Further, we hypothesized that the graph results would support the “distinct subtypes hypothesis,” showing signature patterns rather than a temporal progression of network changes from minimal atrophy to typical AD.

## Materials and Methods

### Participants

A total of 198 AD patients and 230 healthy controls from the ADNI-1 study were initially included in this study. The ADNI study (http://adni.loni.usc.edu/, PI Michael M. Weiner) was launched in 2003 by the National Institute on Aging, the National Institute of Biomedical Imaging and Bioengineering, the Food and Drug Administration, private pharmaceutical companies, and non-profit organizations (33). The project was established to develop standardized imaging techniques and biomarkers in AD research. Participants whose T1-weigthed data did not pass quality control, presented image processing errors, or visual ratings (see below) were not performed, were excluded (23 AD patients excluded and 40 healthy controls excluded). The remaining individuals were classified into subtypes according to their pattern of brain atrophy as detailed in the next section below. The healthy controls with minimal atrophy were selected for the current study in order to be able to determine how AD affects network topology across a range of atrophy subtypes, from minimal atrophy to widespread typical atrophy. Thus, the final sample included 175 AD patients and 81 healthy controls.

The AD patients and healthy controls were clinically diagnosed following standard procedures, as fully detailed elsewhere (34). Briefly, AD diagnosis was based on the NINCDS-ADRDA and DSM-IV criteria for probable AD, as well as a total Clinical Dementia Rating (CDR) (35) score of ≥0.5. The inclusion criteria for the healthy controls were a mini-mental state examination (MMSE) (36) score between 24 and 30, a total CDR score of 0, and a Geriatric Depression Scale (GDS) (37) score ≤5. Exclusion criteria for both AD and healthy controls were significant neurological or psychiatric illness, significant unstable systemic illness or organ failure, and history of alcohol or substance abuse or dependence. All diagnoses were made without the use of MRI. The study was approved by the institutional review boards of all participating ADNI centers. Written informed consent was obtained from all participants or authorized representatives according to the Declaration of Helsinki. All methods were performed in accordance with the relevant guidelines and regulations.

### Magnetic Resonance Imaging, Visual Rating Scales, and AD Subtypes Based on Patterns of Brain Atrophy

A 3D T1-weighted magnetization-prepared rapid gradient-echo (MPRAGE) sequence was acquired on 1.5T MRI scanners (voxel size 1.1 × 1.1 × 1.2 mm^3^) in all participants (33).

Three visual rating scales were applied to the T1-weigthed images to measure regional brain atrophy, as previously described (34). Briefly, medial temporal atrophy (MTA) was assessed with the Scheltens' scale (38), cortical posterior atrophy (PA) with the Koedam's scale (39), and atrophy in the frontal lobe with the global cortical atrophy scale—frontal subscale (GCA-F) (40). Reliability (weighted κ) for the visual ratings in 120 random cases was: Intra-rater (L.C.): MTA-left = 0.94, MTA-right = 0.89, PA = 0.88; GCA-F = 0.83; Inter-rater (L.C. *vs*. rater 2): MTA-left = 0.71, MTA-right = 0.70; PA = 0.88, GCA-F = 0.79. Raters were blinded to patient information and each other's ratings.

**Figure 1:**
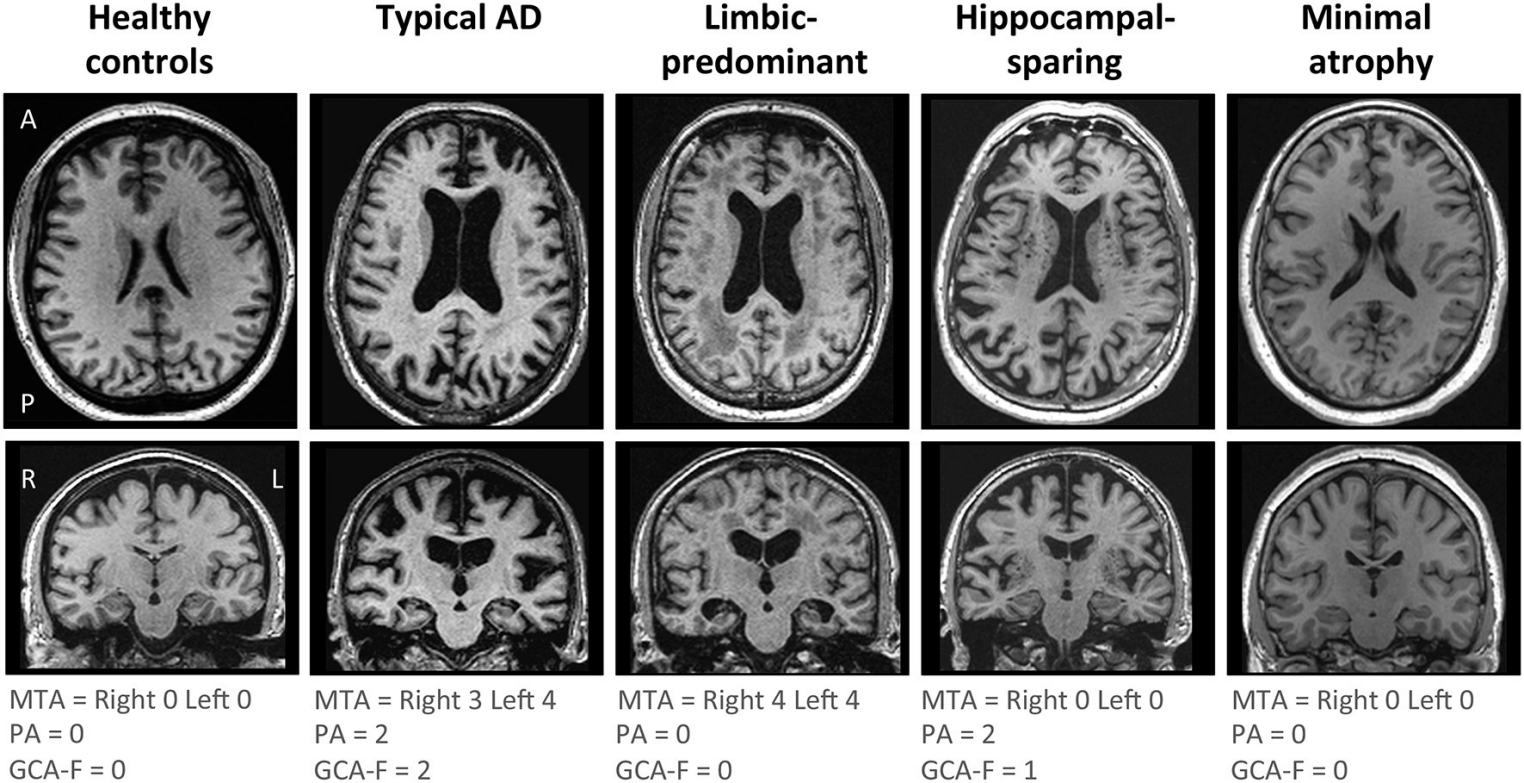
Visual examples of the brain atrophy patterns in the different AD subtypes. Atrophy patterns were determined based on the combination of MTA, PA, and GCA-F visual rating scales. In the three visual rating scales, a score of zero denotes no atrophy, whereas scores from one to three (PA and GCA-F) or four (MTA) indicate an increasing degree of atrophy. Typical AD was defined as abnormal MTA together with abnormal PA and/or abnormal GCA-F. Limbic-predominant was defined as abnormal MTA alone with normal PA and GCA-F. Hippocampal-sparing included abnormal PA and/or abnormal GCA-F, but normal MTA. Minimal atrophy AD was defined as normal scores in MTA, PA, and GCA-F. The figure shows examples for each AD subtype and the healthy controls. A, anterior part of the brain; AD, Alzheimer's disease; GCA-F, global cortical atrophy scale–frontal subscale; L, left; MTA, medial temporal atrophy scale; P, posterior part of the brain; PA, posterior atrophy scale; R, right.

AD subtyping was based on the combination of MTA, PA, and GCA-F using recently proposed cut-offs (34), as previously described (12). Briefly, typical AD was defined as abnormal MTA together with abnormal PA and/or abnormal GCA-F. Limbic-predominant AD was defined as abnormal MTA with normal PA and GCA-F. Hippocampal-sparing AD included abnormal PA and/or abnormal GCA-F, but normal MTA. Minimal atrophy AD displayed normal scores in MTA, PA, and GCA-F. Examples of the different subtypes and their respective MTA, PA, and GCA-F scores can be found in Figure 1.

### Automated Image Processing and Network Construction

TheHiveDB Database system (41) was used to automatically process the T1-weigthed images with FreeSurfer 5.3.0, following standard procedures (42). This provides thickness estimations for cortical regions, volume estimations for subcortical structures, and an estimation of the total intracranial volume (TIV). Quality control was performed both on the original T1-weighted images (43) and the FreeSurfer output.

**Figure 2:**
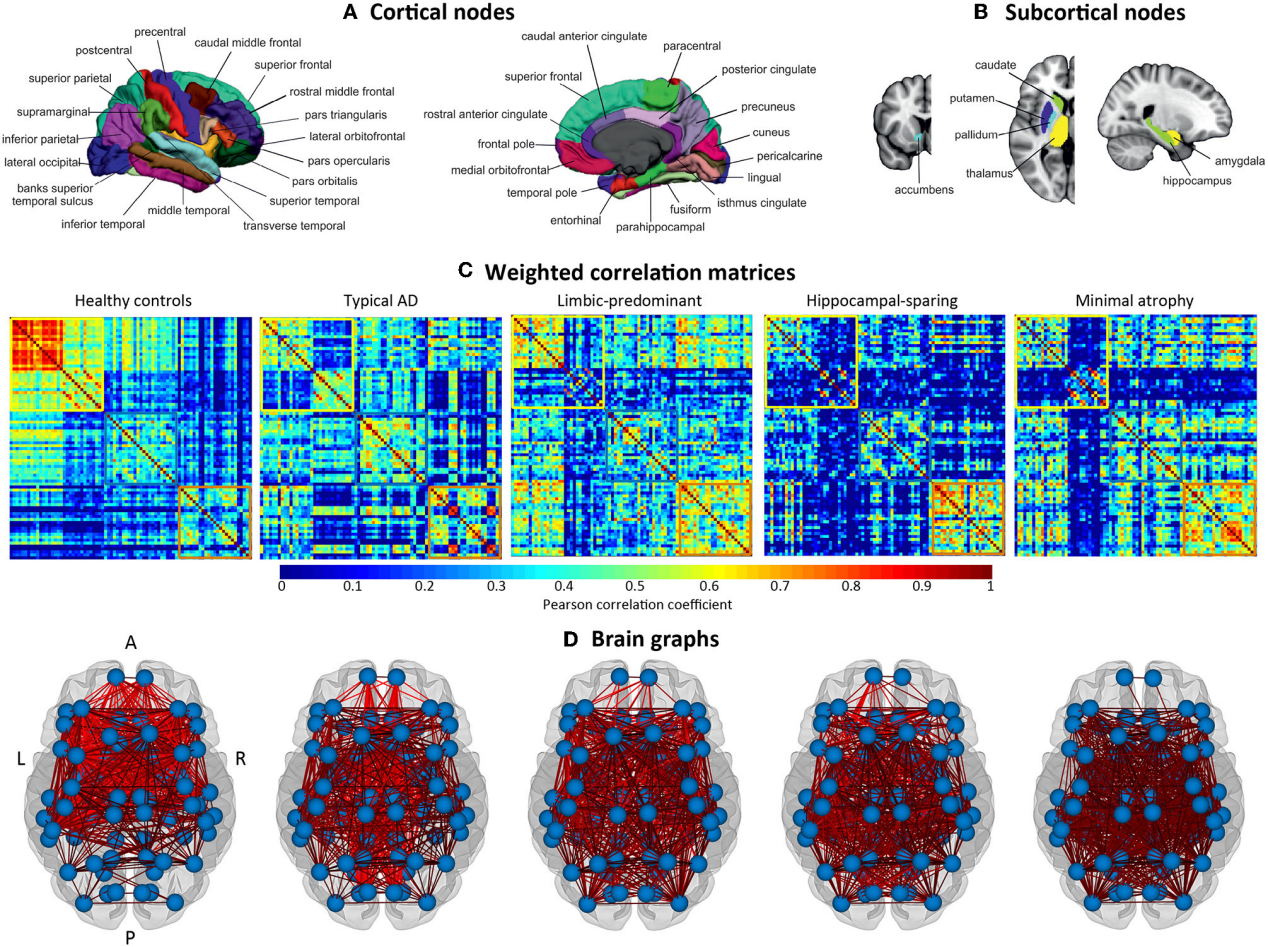
Structural brain networks. A, anterior part of the brain; AD, Alzheimer's disease; L, left; P, posterior part of the brain; R, right. **(A)** Brain regions included as cortical nodes; **(B)** brain regions included as subcortical nodes; **(C)** weighted correlation matrices by study group; **(D)** brain graphs by study group.

The cortical thickness from 68 cortical regions included in the Desikan et al. (44) atlas, and the volumes of the hippocampus, amygdala, thalamus, caudate, putamen, accumbens, and pallidum from the Fischl et al. (45) atlas were selected as nodes for network construction (82 nodes in total, Figures 2A,B). These anatomical measures were corrected by age, sex, and years of education (plus TIV only for subcortical volume estimations) using multiple linear regression. The edges between the nodes were calculated through group-specific association matrices of Pearson correlation coefficients from each pair of nodes (Figure 2C). The matrices were binarized by thresholding the correlation coefficients at a range of network densities (min = 5% to max = 40%, in steps of 1%. Figure 2D shows the resulting brain graphs at the median density of 22%). Network topologies were compared across this range, making sure that disconnected networks and random topologies were excluded from the analysis. Both self-connections and negative correlations were excluded.

Once the networks were constructed, different global and nodal measures were calculated. Nodal measures refer to each specific node whereas global measures refer to the average between all the nodes. Nodal measures are calculated first and then corresponding global measures are calculated by averaging all the nodes across the whole network. For each specific node, global connectivity features (connections with the rest of the network) as well as local connectivity features (connections with the immediate neighbors) can be computed. The following global measures were calculated: *the transitivity* [fraction of a node's neighbors that are also neighbors of each other in the whole network, normalized by the whole network, reflecting how well the nodes are connected to nearby regions forming cliques. The *transitivity* is similar to the commonly used *clustering coefficient* but is less vulnerable to methodological issues such as edge definition, network size, and groups composition (46, 47)], *the modularity* (the extent to which a network can be divided into communities of regions with a large number of within-modules connections and a minimal number of between-module connections), *the average global efficiency* (the average inverse shortest path length between a node and the rest of the network, which, in contrast to the characteristic path length, can be meaningfully computed on disconnected networks), and *the average local efficiency* (similar to the global efficiency but restricted to a given node and its immediate neighbors). The following nodal measures were calculated: *the nodal global efficiency* (for a specific node, the average inverse shortest path length between that node and the rest of the network) and *the nodal local efficiency* (similar to the nodal global efficiency but restricted to a specific node and its immediate neighbors).

**Figure 3:**
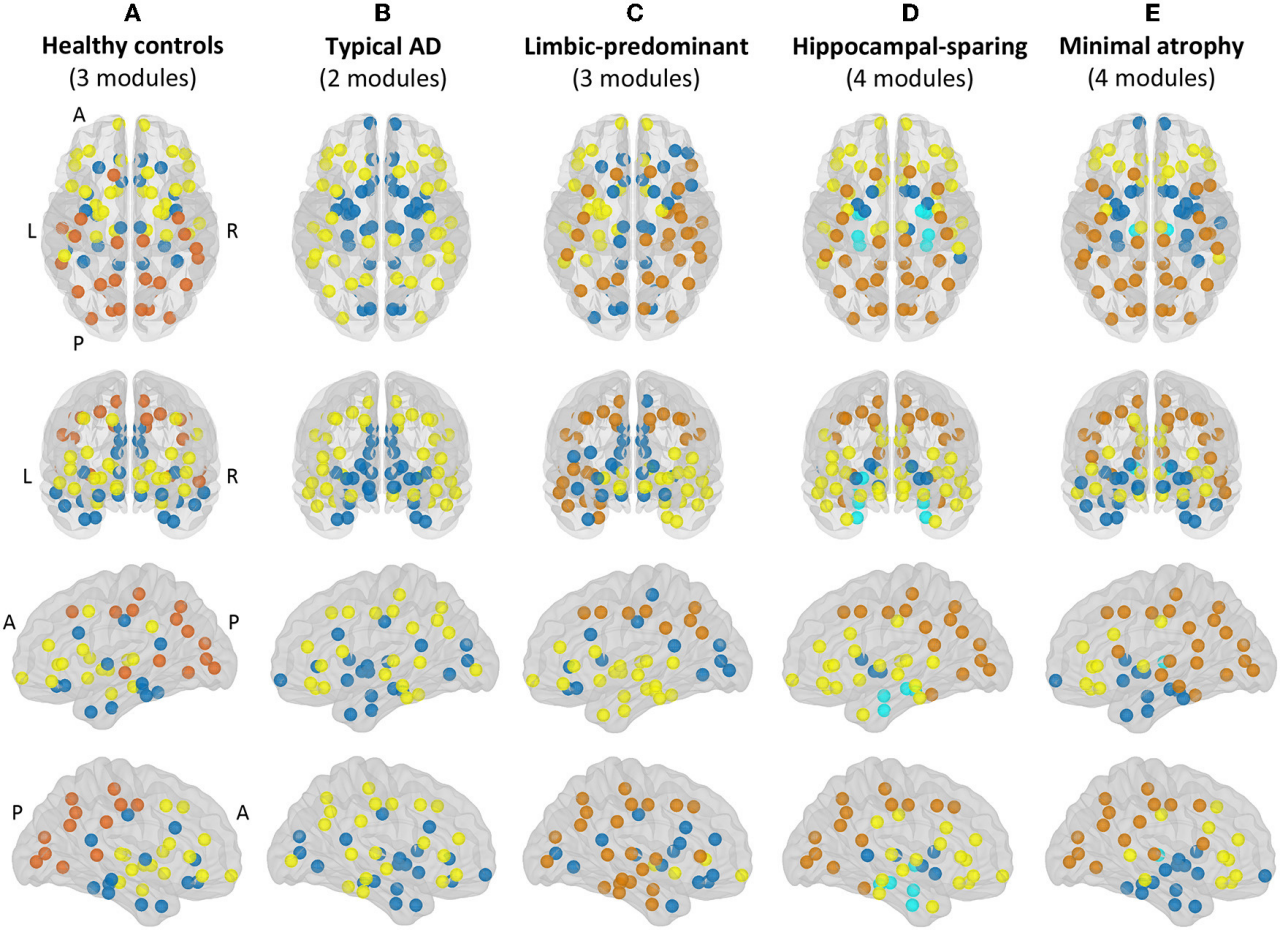
Modules. Module I in yellow, module II in dark blue, module III in orange, module IV in light blue. A, anterior part of the brain; AD, Alzheimer's disease; L, left; P, posterior part of the brain; R, right. **(A)** Healthy controls; **(B)** Typical AD patients; **(C)** Limbic-predominant AD patients; **(D)** Hippocampal-sparing AD patients; **(E)** minimal atrophy AD patients.

Modular analyses were also conducted by applying the Louvain algorithm (48) on weighted networks (i.e., the correlation matrices before binarization) with a gamma value of 1. This method is alternative to the modularity measure explained above. While the modularity is a sophisticated quantitative measure that reflects the existence of communities of regions within a network (29), it cannot provide any information about the specific belonging of brain regions to the actual communities. This can in turn be qualitatively assessed by modular analyses as shown in Figure 3.

The formulae used to calculate all these graph measures are provided by Rubinov and Sporns (26). Network construction, measures calculation, and graph analyses were performed using BRAPH (www.braph.org) (49).

### Demographic and Clinical Variables

Age, sex, and years of education were included for the demographic description of the groups. Clinical severity was assessed with the CDR (35) scale and global cognition with the MMSE (36). Age at disease onset, disease duration, and APOE ε4 status were also included. CSF samples were available for 91 AD patients and 40 healthy controls. Complete procedure descriptions are available at http://www.adni-info.org/.

### Statistical Analysis

ANOVA and the Kruskal-Wallis test were used for between-group comparisons of continuous and dummy demographic and clinical variables. *P*-values in all *post-hoc* analyses were adjusted with the Hochberg's (50) correction for multiple comparisons. Model assumptions were tested in all the cases by visual inspection of data distribution, as well as by inspecting the pertinent statistical parameters. Results were considered significant when *p* ≤ 0.05 (two-tailed). Between-group comparisons of graph measures were conducted through 1000 nonparametric permutations at a range of network densities (min = 5% to max = 40%, in steps of 1%). The 95% confidence intervals of each distribution were used as critical values for testing of the null hypothesis at *p* ≤ 0.05 (two-tailed). The false discovery rate (FDR) correction (51) for multiple comparisons was used at p≤0.05 (two-tailed) on the nodal measures at the median density (22%). All statistical analyses were conducted using SPSS v22, R, and BRAPH.

## Results

The main demographic and clinical characteristics of the study groups are shown in Table 1. Groups mainly differed in age and age at onset. In addition, the AD patients showed as expected lower MMSE scores, higher prevalence of the APOE ε4 allele, and higher prevalence of abnormal CSF biomarker levels as compared with the healthy controls.

**Table 1:** Characteristics of the AD subtypes and healthy controls.

	**Healthy controls (*n* = 81)**	**AD subtypes**				
		**Typical AD (*n* = 89, 50.9%)**	**Limbic-predominant (*n* = 30, 17.1%)**	**Hippocampal-sparing (*n* = 29, 16.6%)**	**Minimal atrophy AD (*n* = 27, 15.4%)**	***p*-value**
Age	74.8 (5.0)	77.8 (6.5)^a^	73.4 (6.1)^b^	78.0 (8.5)^c*^	69.2 (7.1)^a^^,^^b^^,^^d^	<0.001
Sex, % female	57	37	57	52	74^b^	0.006
Years of education	15.8 (3.0)	15.1 (3.3)	14.4 (2.2)	15.6 (2.8)	13.6 (3.9)^a^	0.012
MMSE^e^	29.1 (1.1)	23.0 (2.1)^a^	23.2 (1.9)^a^	23.6 (2.2)^a^	24.1 (1.5)^a^^,^^b^	<0.001
**CDR total**^**e**^						
Score 0, %	100	–	–	–	–	<0.001
Score 0.5, %	–	40	67	57	67	
Score 1, %	–	60	33	43	33	
Age at onset^f^	–	73.8 (7.2)	69.7 (5.7)	75.8 (8.0)^c^	66.7 (7.4)^b^^,^^d^	<0.001
Disease duration^f^	–	3.7 (2.5)	3.4 (2.2)	3.1 (2.9)	2.7 (1.5)	0.324
APOE status, % ε4 allele	22	69^a^	73^a^	52^a^	70^a^	<0.001
CSF Aß_1−42_, % abnormal^g^	33	98^a^	94^a^	83^a^	91^a^	<0.001
CSF T-tau, % abnormal^h^	18	55^a^	78^a^	78^a^	64^a^	<0.001
CSF p-tau, % abnormal	52	78^a^	87^a^	79^a^	82^a^	<0.001

*The table shows mean (SD) except for sex, CDR scores, APOE status, and the CSF biomarkers, where percentage is reported*.

a Significantly different from healthy controls;

b Significantly different from typical AD;

c Significantly different from limbic-predominant;

d *Significantly different from hippocampal-sparing*.

c* *p = 0.054*.

e N = 244;

f *N = 137*;

g N = 131;

h *N = 130*.

*AD = Alzheimer's disease; APOE, apolipoprotein E; Aß_1-42_, amyloid-ß-peptide 1-42; CDR, clinical dementia rating; CSF, cerebrospinal fluid; MMSE, mini-mental state examination; p-tau, level of phosphorylated tau protein; T-tau, total level of tau protein*.

### Global Network Analysis

**Figure 4:**
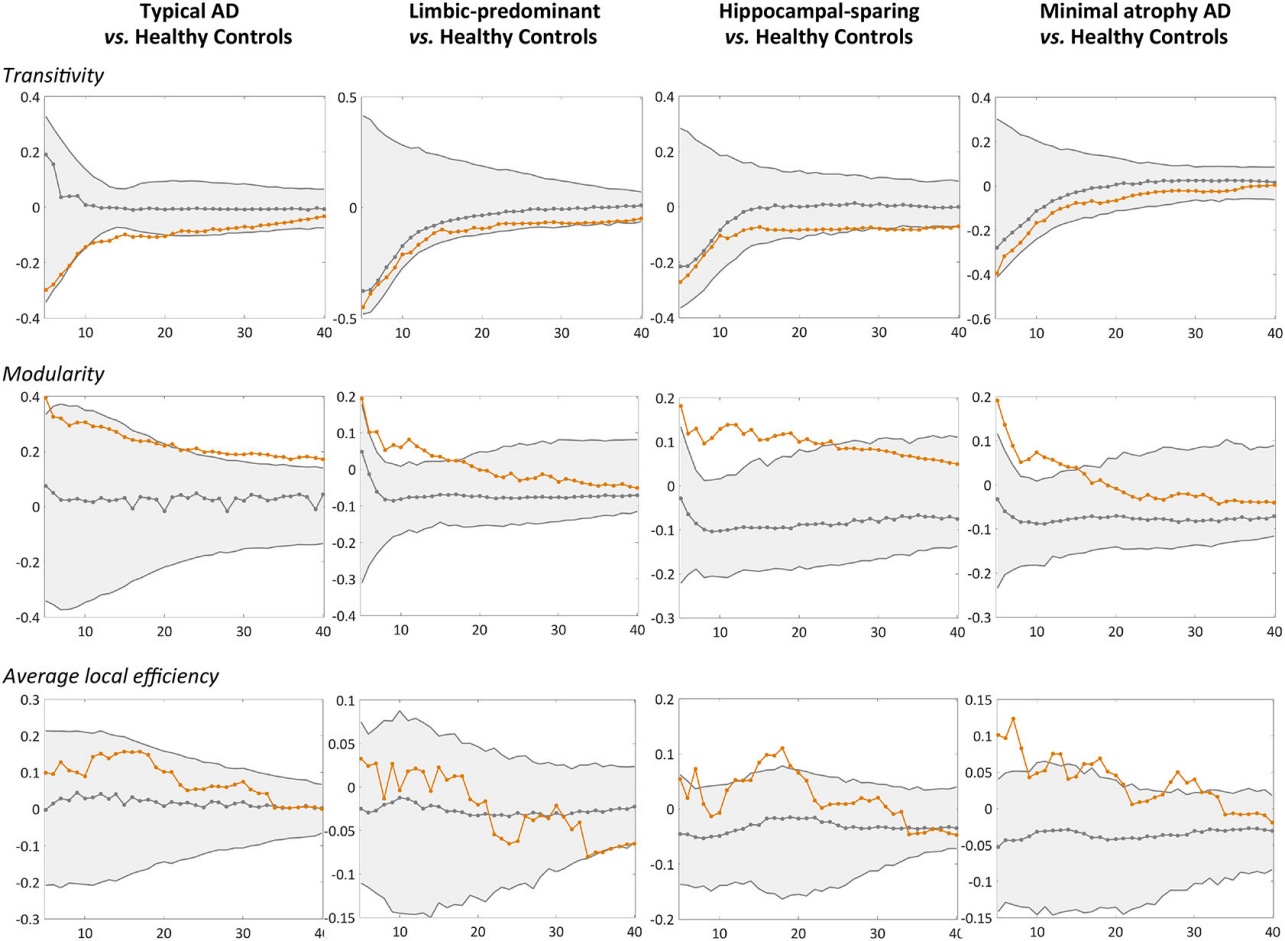
Comparison of the AD subtypes with the healthy controls in global network measures. Network densities are displayed on the x-axis from min = 5% to max = 40%, in steps of 1%. Between-group differences in the global graph measures are displayed on the y-axis. AD, Alzheimer's disease.

When comparing the AD subtypes with the healthy controls, we found that the modularity was increased in all the AD subtypes (Table 2A, Figure 4). The transitivity was decreased in typical and hippocampal-sparing AD. The average local efficiency was increased in hippocampal-sparing and minimal atrophy AD, whereas no differences were observed in the average global efficiency.

**Table 2:** Summary of the global and nodal network results.

**Measure**	**Healthy controls *vs*. Typical AD**	**Healthy controls *vs*. Limbic-predominant**	**Healthy controls *vs*. Hippocampal-sparing**	**Healthy controls *vs*. Minimal atrophy AD**
**(A) GLOBAL MEASURES**				
Transitivity	↓	–	↓	–
Modularity	↑	↑	↑	↑
Average global efficiency	–	–	–	–
Average local efficiency	–	–	↑	↑
**(B) NODAL MEASURES**				
**Nodal global efficiency**				
*Two-tailed t-test*	↑ 5 regions ↓ 1 region	–	–	–
*One-tailed t-test*	↑ 10 regions ↓ 3 regions	↑ 2 regions ↓ 1 region	↑ 3 regions	↑ 9 regions
**Nodal local efficiency**				
*Two-tailed t-test*	↑ 3 regions ↓ 1 region	↓ 2 regions	–	–

*Results were considered significant when p ≤ 0.05 (two-tailed). Since trends to significant differences were observed in nodal measures in both hippocampal-sparing and minimal atrophy AD, FDR-corrected one-tailed t-tests were conducted showing significant results in nodal global efficiency (displayed in the table). The one-tailed t-tests were also conducted for typical and limbic-predominant AD, and the results are displayed in the table for completeness of information. AD, Alzheimer's disease; ↑, higher values in AD patients; ↓, lower values in AD patients; −, non-significant results*.

### Nodal Network Analysis

**Figure 5:**
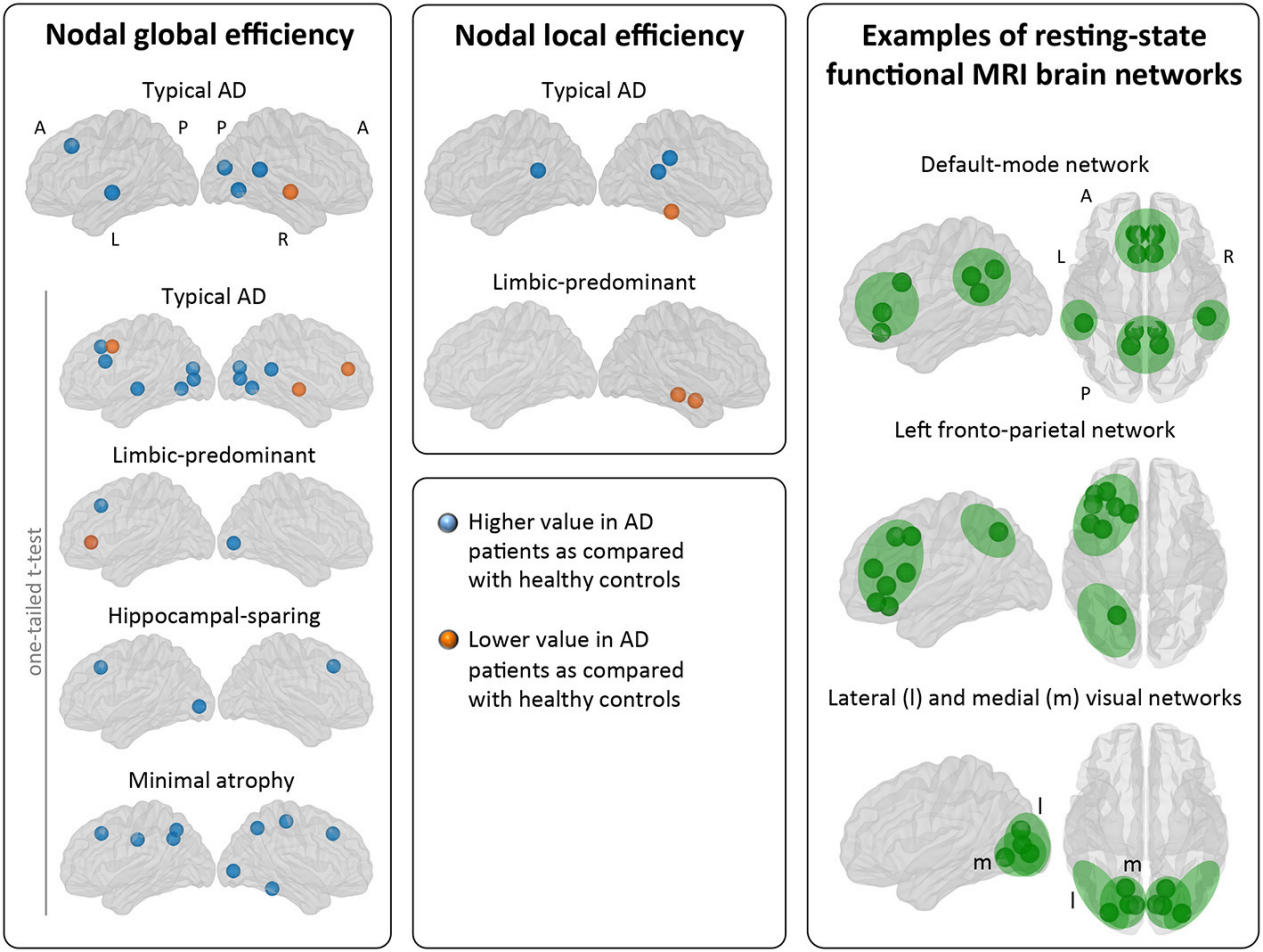
Comparison of the AD subtypes with the healthy controls in nodal network measures. The right box shows examples of resting-state functional MRI brain networks for interpretation of the nodal results obtained in the current study. Our graph nodes were assigned to the default-mode, fronto-parietal, and visual networks according to a previous review (52). AD, Alzheimer's disease; A, anterior; l, lateral; L, left; m, medial; MRI, magnetic resonance imaging; P, posterior; R, right.

When comparing the AD subtypes with the healthy controls, typical AD showed significant differences in the nodal global efficiency and nodal local efficiency (Tables 2B, 3, Figure 5). The nodal global efficiency was increased in the left superior frontal and temporal cortex, and in medial and lateral regions of the right posterior cortex. In contrast, the nodal global efficiency was decreased in the right superior temporal gyri. The nodal local efficiency was increased in medial and lateral posterior regions, while it was decreased in the right inferior temporal gyri.

**Table 3:** Nodal network measures.

**Region**	**Brain network**	**Healthy controls**	**Typical AD**	**FDR-corrected *p*-value**
**Nodal global efficiency**				
Left superior frontal	F-P (also S, DA/VA)	0.345	0.667	<0.001
Left superior temporal	A	0.311	0.603	<0.001
Right superior temporal	A	0.726	0.607	<0.001
Right isthmus cingulate	DMN	0	0.657	<0.001
Right cuneus	MV	0	0.604	<0.001
Right lingual	MV	0	0.627	<0.001
**Nodal local efficiency**				
Left isthmus cingulate	DMN	0	0.891	0.002
Right isthmus cingulate	DMN	0	0.756	0.002
Right inferior temporal	LV	0.956	0.500	0.002
Right supramarginal	DMN	0.521	0.820	0.002
		**Healthy controls**	**Limbic- predominant**	
**Nodal local efficiency**				
Right hippocampus	DMN	0.839	0.300	<0.001
Right amygdala	DMN	0.909	0	<0.001

*A, auditory network; AD, Alzheimer's disease; DA, dorsal attention network; DMN, default-mode network; F-P, fronto-parietal network; LV, lateral visual (also known as secondary visual); MV, medial visual (also known as primary visual); S, salience network; VA, ventral attention network*.

Interestingly, limbic-predominant showed decreased nodal local efficiency in the hippocampus and amygdala (Tables 2B, 3, Figure 5).

No significant differences with the healthy controls were observed in hippocampal-sparing and minimal atrophy AD when using two-tailed *t*-tests. However, multiple trends for significance were observed and FDR-corrected follow-up analyses using one-tailed t-tests were thus conducted. These tests revealed that hippocampal sparing had increased nodal global efficiency in the superior frontal cortex and the left lateral occipital cortex (Table 2B, Supplementary Table 2, Figure 5). Minimal atrophy AD had increased nodal global efficiency in the superior frontal cortex and several medial and lateral regions of the posterior cortex (Table 2B, Supplementary Table 2, Figure 5).

### Weighted Correlation Matrices

The weighted correlation matrices are displayed in Figure 2C (Please see Supplementary Figures 1–5 for matrices with larger size and labeled regions).

Visual inspection of the matrices reveals that the frontal and subcortical gray matter regions were strongly correlated in the healthy controls, both bilaterally with their homologous regions and ipsilaterarly with each other (e.g., frontal and subcortical regions with each other) (Supplementary Figure 1).

In contrast, different correlation patterns were observed in the AD subtypes. Overall, the pattern of correlations was more dedifferentiated (less segregated) in limbic-predominant and minimal atrophy AD. In typical AD, medial regions of the frontal, parietal, and occipital cortex were strongly correlated, both bilaterally and ipsilaterarly (Supplementary Figure 2). In limbic-predominant AD, lateral regions of the temporal cortex were strongly correlated ipsilaterarly, and moderate correlations between regions diffusively located across the cortex were also observed, both bilaterally and ipsilaterarly (Supplementary Figure 3). In hippocampal-sparing AD, lateral and medial regions of the parietal and occipital cortex were strongly correlated, both bilaterally and ipsilaterarly (Supplementary Figure 4). In minimal atrophy AD, lateral regions of the parietal cortex were strongly correlated, both bilaterally and ipsilaterarly (Supplementary Figure 5).

### Brain Modules

The correlation patterns described above led to distinct modular topology in the different groups, as shown in Figure 3 and Supplementary Table 1.

Three modules were identified in the healthy controls (Figure 3A). Module I included lateral frontal areas and subcortical gray matter structures. Module II included the orbitofrontal cortex, the cingulate cortex (both anterior and posterior), medial and lateral regions of the temporal cortex, and the insula. Module III mostly included the posterior cortex, extending to the premotor cortex, the left superior frontal gyri, and the banks of the superior temporal cortex.

Different modular organizations were identified in the AD subtypes. Typical AD only showed two modules (Figure 3B). In module I, the frontal cortex lost its modular connectivity with the subcortical gray matter structures, and instead, clustered together with lateral regions of the temporal, parietal, and occipital cortex. Module II included most of the medial regions, similar to limbic-predominant AD, but also included all the subcortical structures.

Limbic-predominant AD was the only subtype displaying modular asymmetry (Figure 3C). The posterior cortex module (module III) occupied regions of the lateral frontal cortex, and extended to many regions of the temporal cortex, but only on the right hemisphere. On the left hemisphere, a totally new modular configuration emerged with module I being the most prominent and occupying frontal, temporal, and subcortical regions. Module II is diffuse and mainly included medial regions.

Hippocampal-sparing AD displayed four modules (Figure 3D). Interestingly, the regions of the medial temporal cortex clustered together (module IV). The posterior cortex module (module III) occupied regions of the lateral frontal cortex. Several subcortical gray matter structures clustered together (module II), resulting in modular disconnection with the frontal cortex (module I).

Finally, minimal atrophy AD displayed three modules organized in a rather similar manner as in the healthy controls (Figure 3E), but the right and left thalamus emerged as a fourth new and separate module (module IV). Also, the posterior cortex module (module III) occupied more regions of the lateral frontal and temporal cortex than in the healthy controls. Thus, subcortical gray matter structures clustered together with the remaining temporal regions (module II), resulting in modular disconnection with the frontal cortex (module I).

## Discussion

This is the first study assessing network topology in different AD subtypes, to the best of our knowledge. Although signs of disconnection were observed, the affected networks were fairly different, matching to a large extent the regional pattern of brain atrophy that defines each subtype. For instance, the hippocampus and amygdala resulted disconnected from their neighboring regions in limbic-predominant AD, presumably related to the characteristic regional atrophy and NFT deposition in the medial temporal lobe in this subtype. In typical AD, the frontal, temporal, parietal, and occipital regions were disconnected from long-distance regions, presumably related to the characteristic widespread atrophy pattern and NFT deposition in this subtype. Furthermore, network abnormalities were detected in the absence of overt brain atrophy in minimal atrophy AD. Network abnormalities also extended beyond the patterns of regional brain atrophy, clearly showing that the disease is expressed differently across the four investigated subtypes. Below we discuss our findings more in detail.

The modular organization seen in the healthy controls was lost in the four subtypes, but each subtype evidenced its own signature reorganization, leading to disconnection of different brain areas. The medial temporal lobe was found to be isolated from the frontal and parietal association cortex both in hippocampal-sparing and minimal atrophy AD. This finding could be the basis for the cognitive results previously reported in the same cohort, where reduced performance in traditionally frontal and parietal cognitive functions was associated with reduced memory in these two subtypes (12). On the contrary, impairment in learning is prominent and sufficient to produce impaired delayed recall in typical and limbic-predominant AD. Hence, traditionally frontal and parietal cognitive functions lack of a central contribution to the memory profile in these two subtypes (12). Our modular analyses showed that, indeed, regions of the medial temporal lobe clustered together with the frontal and parietal association areas of the left hemisphere in both typical and limbic-predominant AD. Thus, disruption of this large network cannot be the explanation for memory impairment in typical and limbic-predominant AD, in contrast to hippocampal-sparing and minimal atrophy AD, but the devastation of the medial temporal lobe may be.

Together with these changes in the modular organization, the modularity measure was increased in the four subtypes, further demonstrating that the brain connectome tends to get fragmented into small isolated modules in the four AD subtypes. Increased modularity has frequently been reported in previous AD studies (49, 53). The novelty of our finding is the different module reorganization shown by each subtype, in part likely reflecting the differential spread of NFT and subsequent regional atrophy in these subtypes (1, 23).

Other novel findings are the changes observed in global segregation measures across the subtypes, which contrasted with the lack of changes in global integration measures (54). In particular, we detected abnormalities in the transitivity and average local efficiency measures, but not in the average global efficiency. Transitivity was decreased in typical and hippocampal-sparing AD. In contrast, the average local efficiency was increased in minimal atrophy AD and hippocampal-sparing. Decreases in transitivity have previously been reported in AD (46, 49, 53), suggesting the loss of connections between neighboring regions. Increased average local efficiency can be interpreted as a compensatory brain response (49). The involved regions might increase their number of connections with the closest neighbors, forming new paths to continue transferring information along the network. This could result in a more segregated network that looses specificity and effectiveness (55). However, increased average local efficiency could also be interpreted as a sign of neighboring regions sharing the same pathological mechanism, which is justified by the assumption that the regions degenerate at the same rate and thus co-vary with each other (22, 23).

Our results on nodal measures offer an interesting glimpse on the regional signature of each subtype. Areas of the posterior cingulate/precuneus showed increased efficiency measures in both typical and minimal atrophy AD. The posterior cingulate/precuneus is a key area of the default-mode network that underpins episodic memory, semantic processing, and attention by connecting medial frontal, lateral parietal, and medial temporal regions (56). In typical AD, an increase in nodal local efficiency was also observed in the right supramarginal gyri, another region of the default-mode network. Increases in local efficiency has previously been reported in AD (49). These increases could thus reflect a shared neurodegeneration process in the whole default-mode network (23, 57), which could explain the impact of impaired semantic processing and attention in memory performance reported in these two subtypes, i.e., typical and minimal atrophy AD (12). We also observed network abnormalities in medial temporal regions belonging to the default-mode network. The nodal local efficiency measures indicated abnormal connections of medial temporal regions with their close neighbors in limbic-predominant AD, presumably reflecting the devastation of the temporal cortex in this subtype (2–6, 12, 18). Thus, cortical areas of the default-mode network were clearly involved in typical, limbic-predominant, and minimal atrophy AD. Disruption of the default-mode network is indeed a consistent finding in studies of heterogeneous groups of AD patients (56). Actual disconnection of the default-mode network in typical, limbic-predominant, and minimal atrophy AD should be investigated in future studies including functional MRI data.

In addition, changes in the nodal measures extended beyond cortical areas of the default-mode network in the four subtypes. Nodal global efficiency was increased in parietal areas in minimal atrophy AD, and in occipital areas in the four subtypes. These areas are part of the fronto-parietal, dorsal attention, sensory-motor, and lateral visual brain networks. Nodal global efficiency was also mostly increased in frontal areas in all the subtypes, although it was decreased in the middle frontal cortex in typical AD. These areas are part of the fronto-parietal, salience, executive control, and ventral/dorsal attention brain networks. Both increases and decreases in typical AD, and only increases in minimal atrophy AD, were observed in efficiency measures in the temporal lobe. The affected areas are part of the auditory and lateral visual brain networks. We should note that some of the regions discussed in these paragraphs may be part of more than one network (please see Table 3 and Supplementary Table 2). However, our current results in connection with the atrophy patterns and the clinical and cognitive profiles previously described (1–3, 11, 12, 58) suggest that the main involved networks seem to be the default-mode, fronto-parietal, and visual networks. Functional MRI studies are warranted to further confirm this interpretation. We also observed that differences in nodal global efficiency involved many more regions in minimal atrophy AD than in limbic-predominant and hippocampal-sparing AD. An explanation for this is that given similar clinical severity across these subtypes, more extensive network abnormalities may be needed to give the clinical symptoms in minimal atrophy AD in the absence of overt brain atrophy. Lower cognitive reserve (13) and small vessel disease in strategic white matter areas (15) could increase network vulnerability to more intense tau-related pathology and neurodegeneration in minimal atrophy AD (59), which as previously been demonstrated through elevated CSF total and phosphorylated tau levels (12, 15).

The signature findings on the nodal measures could have a biological interpretation related to different networks involved in these subtypes. We hypothesize that changes in typical and minimal atrophy AD are mostly related to the default-mode, fronto-parietal, and visual networks. However, changes in limbic-predominant AD would be mostly related to the default-mode network, and changes in hippocampal-sparing could be entirely confined to the fronto-parietal and visual networks. Especially the default-mode and the fronto-parietal networks include long and dense but poorly myelinated axons (60). Their central involvement in high-order processing, as well as their constant exposure to high energy demands and oxidative stress (60), makes them especially vulnerable to pathology (22, 56, 57). Thus, these networks may be the conduits used by the NFT to spread differentially in the four subtypes, leading to the distinct patterns of atrophy observed in sMRI data.

The changes in the segregated graph measures discussed above could well be the mechanism behind the impairment of segregated cognitive functions (e.g., learning of episodic memory and semantic abilities), rather than integrated cognitive functions (e.g., executive functions, processing speed, attention) in AD. Indeed, impairment of segregated cognitive functions such as learning of episodic memory is an early event and a hallmark of AD (61, 62). This may be a relevant finding to explain the memory impairment seen in hippocampal-sparing and, specially, minimal atrophy AD, an explanation that remained elusive until recently given their characteristic patterns of atrophy (or no atrophy) (12). Integrated cognitive functions could also be affected as a consequence of their segregated cognitive components being primarily affected (e.g., lexical access after disruption of the semantic system), but this would happen later in the disease. For instance, impairment of integrated cognitive functions such as executive functioning is common in the advanced stages of AD. Notwithstanding, executive dysfunction could also be an early symptom before memory impairment in the atypical executive presentation of AD (62, 63). This is presumably explained by the loci of atrophy in this presentation, with involvement of the frontal lobe (40, 61). Therefore, changes in segregated graph measures may also explain the executive dysfunction previously seen in hippocampal-sparing AD (1–3, 11, 12, 58), due to atrophy in the frontal lobe and/or disconnection with the posterior cortex.

The clinical implications of these findings are important. Cognitive interventions based on compensation and substitution brain mechanisms are commonly used in patients with memory impairment. In compensation mechanisms, the original brain network is partially retained and alternative brain regions are recruited for its rescue. In substitution mechanisms, the original brain network is no longer functional and alternative brain regions are recruited to enable a new anatomical-functional system. In practice, compensation has potential when the patient retains some learning and encoding capacity. Substitution is needed when this capacity is mostly lost. Our current data indicates that compensation strategies should be primarily used in hippocampal-sparing and minimal-atrophy, because the learning and encoding capacity is partially spared (12). Indeed, a study performed in the same cohort showed that hippocampal-sparing and minimal-atrophy were the subtypes getting greatest benefit from additional help when retrieving stored information (12). The clinician needs to be careful and explore the compensatory cognitive functions that may work the best for each patient. The data on the disrupted brain networks reported in the current study may be important for this purpose. Cognitive interventions in typical and limbic-predominant AD have however the great challenge of finding a good substitute of the devastated medial temporal lobe. The strategy should thus be substitutive, for instance, by training other memory systems such as procedural memory.

Some limitations of the current study should be mentioned. All our AD patients fulfilled the amnestic criteria at entry and factors such as vascular pathology were excluded. Our results should thus be replicated in a more heterogeneous clinical sample that also includes non-amnestic AD presentations. AD patients in the ADNI are clinically diagnosed and, among those with CSF data, a very small proportion (7%) are amyloid negative in our current study. We decided not to exclude amyloid negative AD patients because that would lead to reduced size of some of the subtypes. Future studies should thus recruit larger groups and focus only on amyloid positive AD patients. Investigating the relationship between APOE genotype and network topology in larger subtype groups is also warranted. Although 1.5T MRI data have some limitations in comparison with 3T MRI data, we selected 1.5T MRI data because the size of this subsample in the ADNI-1 cohort is appropriate for our aims, while the 3T MRI data is very limited. Although cross-sectional data can provide an important insight on pathology spread (22, 23), longitudinal analyses are warranted to complement our current findings. Likewise, our interpretations on pathology spread should be substantiated with tau-PET data. Group-level analysis is the most common form of studying network topology using structural MRI data. However, future work should explore methods that can generate individual networks (64), so that correlations between network measures and cognitive and clinical measures can be performed in the different subtypes.

In conclusion, we demonstrated distinct signature patterns of network disruption, which parallel the atrophy patterns that define the four AD subtypes and, interestingly, extend also to other brain regions presumably reflecting the spread of NFT before overt brain atrophy can be detected in those regions. The four AD subtypes presented network changes consistent with the isocortical NFT stage (stage V) of the Braak and Braak scheme (17), in which pathology occupies most of the neocortical association areas, although largely sparing the primary somatosensory and motor cortex. Thus, our findings support the “distinct subtypes hypothesis,” with pathology spreading through the brain in a different manner in these subtypes, as opposed to the “staging hypothesis” (1, 8, 12). We hope that our current findings can promote personalized medicine approaches in the short term by guiding tailored cognitive interventions, and help characterizing more homogeneous AD groups for drug discovery in the future.

## Ethics Statement

The study was approved by the institutional review boards of all participating ADNI centers. Written informed consent was obtained from all participants or authorized representatives according to the Declaration of Helsinki. All methods were performed in accordance with the relevant guidelines and regulations.

## Author Contributions

All the authors contributed to the conception and design of the study. DF organized the database and wrote the first draft of the manuscript. DF and JP performed the statistical analysis. JP, GV, and EW wrote sections of the manuscript. All authors contributed to manuscript revision, read and approved the submitted version.

### Conflict of Interest Statement

The authors declare that the research was conducted in the absence of any commercial or financial relationships that could be construed as a potential conflict of interest.
