# Supplementary material for: Subtypes of Alzheimer's Disease Display Distinct Network Abnormalities Extending Beyond Their Pattern of Brain Atrophy
Source: Front Neurol. 2019 May 28;10:524. doi: 10.3389/fneur.2019.00524 (PMC6547836; doi:10.3389/fneur.2019.00524)
Supplement: Supplementary file 1 [file Data_Sheet_1.pdf]

# SUPPLEMENTARY MATERIAL

**Supplementary Table 1. Brain modules in healthy controls and the subtypes of Alzheimer's disease**

AD = Alzheimer's disease.

| Hemisphere | Brain region | Healthy controls | Typical AD | Limbic-predominant | Hippocampal-sparing | Minimal atrophy AD |
|---|---|---|---|---|---|---|
| Left | Superior frontal | III | I | III | III | III |
| Left | Frontal pole | I | II | I | I | II |
| Left | Rostral middle frontal | I | I | I | I | I |
| Left | Caudal middle frontal | I | I | III | III | III |
| Left | Pars orbitalis | I | I | I | I | I |
| Left | Lateral orbito frontal | II | I | II | I | I |
| Left | Pars triangularis | I | I | I | I | I |
| Left | Pars opercularis | I | I | I | I | III |
| Left | Medial orbito frontal | II | I | II | I | I |
| Left | Rostral anterior cingulate | I | II | II | I | I |
| Left | Caudal anterior cingulate | II | II | II | I | I |
| Left | Insula | II | II | I | I | I |
| Left | Precentral | III | I | III | III | III |
| Left | Postcentral | III | I | III | III | III |
| Left | Supramarginal | I | I | III | III | III |
| Left | Superior parietal | III | I | III | III | III |
| Left | Inferior parietal | III | I | III | III | III |
| Left | Paracentral | III | I | II | III | III |
| Left | Posterior cingulate | II | II | II | I | I |
| Left | Isthmus cingulate | II | II | II | III | III |
| Left | Precuneus | III | I | III | III | III |
| Left | Cuneus | III | II | II | III | III |
| Left | Pericalcarine | III | II | II | III | III |
| Left | Lingual | III | II | II | III | III |
| Left | Lateral occipital | III | I | II | III | III |
| Left | Transverse temporal | I | I | I | I | III |
| Left | Temporal banks | III | I | I | I | III |
| Left | Superior temporal | III | I | I | I | III |
| Left | Middle temporal | II | I | I | I | III |
| Left | Inferior temporal | II | I | I | I | II |
| Left | Temporal pole | II | II | I | I | II |
| Left | Entorhinal | II | II | I | IV | II |
| Left | Parahippocampal | II | II | I | IV | II |
| Left | Fusiform | II | I | I | III | III |
| Left | Thalamus | I | II | I | I | IV |
| Left | Caudate | I | II | II | II | II |
| Left | Putamen | I | II | I | II | II |
| Left | Pallidum | I | II | I | II | II |
| Left | Hippocampus | I | II | I | IV | II |
| Left | Amygdala | I | II | I | IV | II |
| Left | Accumbens | I | II | I | I | II |
| Right | Superior frontal | I | I | III | III | I |
| Right | Frontal pole | I | II | I | I | II |
| Right | Rostral middle frontal | I | I | II | I | I |
| Right | Caudal middle frontal | I | I | III | III | III |
| Right | Pars orbitalis | I | I | II | I | I |

| | | | | | | |
|---|---|---|---|---|---|---|
| Right | Lateral orbito frontal | II | I | II | I | I |
| Right | Pars triangularis | I | I | III | I | I |
| Right | Pars opercularis | I | I | II | I | III |
| Right | Medial orbito frontal | II | II | II | I | I |
| Right | Rostral anterior cingulate | II | II | I | I | I |
| Right | Caudal anterior cingulate | II | II | II | I | I |
| Right | Insula | II | II | III | I | II |
| Right | Precentral | III | I | III | III | III |
| Right | Postcentral | III | I | III | III | III |
| Right | Supramarginal | III | I | III | I | III |
| Right | Superior parietal | III | I | III | III | III |
| Right | Inferior parietal | III | I | III | III | III |
| Right | Paracentral | III | I | III | III | III |
| Right | Posterior cingulate | II | II | II | I | I |
| Right | Isthmus cingulate | II | II | II | III | III |
| Right | Precuneus | III | I | III | III | III |
| Right | Cuneus | III | II | III | III | III |
| Right | Pericalcarine | III | II | II | III | III |
| Right | Lingual | III | II | II | III | III |
| Right | Lateral occipital | III | I | III | III | III |
| Right | Transverse temporal | I | I | III | I | III |
| Right | Temporal banks | III | I | III | II | I |
| Right | Superior temporal | I | I | III | I | II |
| Right | Middle temporal | II | I | III | I | I |
| Right | Inferior temporal | II | I | III | I | II |
| Right | Temporal pole | II | II | II | I | II |
| Right | Entorhinal | II | II | III | IV | II |
| Right | Parahippocampal | II | II | III | IV | II |
| Right | Fusiform | II | I | III | III | II |
| Right | Thalamus | I | II | II | II | IV |
| Right | Caudate | I | II | II | II | II |
| Right | Putamen | I | II | II | II | II |
| Right | Pallidum | I | II | I | IV | II |
| Right | Hippocampus | I | II | III | IV | II |
| Right | Amygdala | I | II | III | IV | II |
| Right | Accumbens | I | II | I | II | II |

**Supplementary Table 2. Results on nodal global efficiency from FDR-corrected one-tailed t-tests**

A = auditory network; AD = Alzheimer’s disease; DA = dorsal attention network; DMN = default-mode network; EC = executive control; F-P = fronto-parietal network; LV = lateral visual (also known ad secondary visual); MV = medial visual (also known as primary visual); S = salience network; S-M = sensory-motor network; VA = ventral attention network.

| Region | Brain network | Healthy controls | Typical AD | FDR-corrected p-value |
|---|---|---|---|---|
| Left superior frontal | F-P (also S, DA/VA) | 0.345 | 0.667 | 0.007 |
| Left cuneus | MV | 0.019 | 0.585 | 0.007 |
| Left pericalcarine | MV | 0.025 | 0.604 | 0.007 |
| Left lingual | MV | 0.360 | 0.653 | 0.007 |
| Left caudal anterior cingulate | DMN (also S, EC, F-P) | 0 | 0.548 | 0.007 |
| Left caudal middle frontal | DMN (also S, EC, F-P) | 0.687 | 0.578 | 0.007 |
| Left superior temporal | A | 0.211 | 0.603 | 0.007 |
| Right rostral middle frontal | F-P (also S, EC, DA/VA) | 0.706 | 0.537 | 0.007 |
| Right superior temporal | A | 0.726 | 0.607 | 0.007 |
| Right isthmus cingulate | DMN | 0 | 0.657 | 0.007 |
| Right cuneus | MV | 0 | 0.604 | 0.007 |
| Right pericalcarine | MV | 0.185 | 0.526 | 0.007 |
| Right lingual | MV | 0 | 0.627 | 0.007 |
| | | **Healthy controls** | **Limbic-predominant** | |
| Left superior frontal | F-P (also S, DA/VA) | 0.345 | 0.724 | <0.001 |
| Left rostral anterior cingulate | DMN (also S, EC, F-P) | 0.610 | 0 | <0.001 |
| Right lateral occipital | LV | 0.314 | 0.702 | <0.001 |
| | | **Healthy controls** | **Hippocampal-sparing** | |
| Left superior frontal | F-P (also S, DA/VA) | 0.345 | 0.711 | <0.001 |
| Left lateral occipital | LV | 0.366 | 0.629 | <0.001 |
| Right superior frontal | F-P (also S, DA/VA) | 0.625 | 0.715 | <0.001 |
| | | **Healthy controls** | **Minimal atrophy AD** | |
| Left superior frontal | F-P (also S, DA/VA) | 0.345 | 0.704 | 0.003 |
| Left posterior cingulate | DMN (also S) | 0.406 | 0.687 | 0.003 |
| Left precuneus | DMN | 0.443 | 0.689 | 0.003 |
| Left superior parietal | F-P (also DA) | 0.438 | 0.663 | 0.003 |
| Right superior frontal | F-P (also S, DA/VA) | 0.625 | 0.710 | 0.003 |
| Right lateral occipital | LV | 0.314 | 0.650 | 0.003 |
| Right paracentral | S-M | 0.437 | 0.673 | 0.003 |
| Right fusiform | LV | 0.530 | 0.698 | 0.003 |
| Right superior parietal | F-P (also DA) | 0.457 | 0.654 | 0.003 |

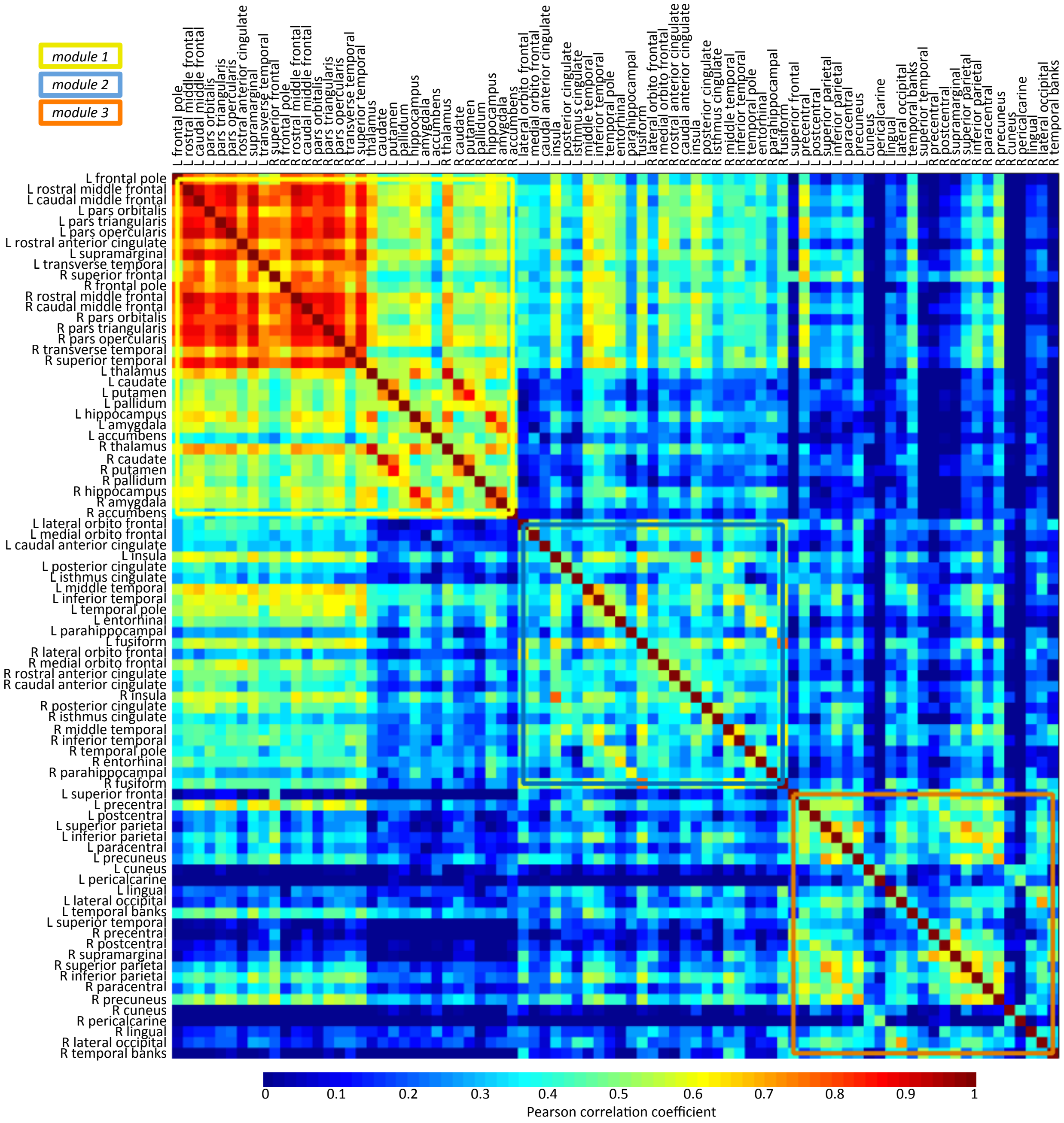


**Supplementary Figure 1. Weighted correlation matrix (healthy controls).** The brain regions were ordered by the brain modules identified in the healthy controls (Table 2 and Fig. 3 in the main text). The yellow square (upper-left corner) indicates the brain regions belonging to module 1. The blue square (center) indicates the brain regions belonging to module 2. The orange square (bottom-right corner) indicates the brain regions belonging to module 3.

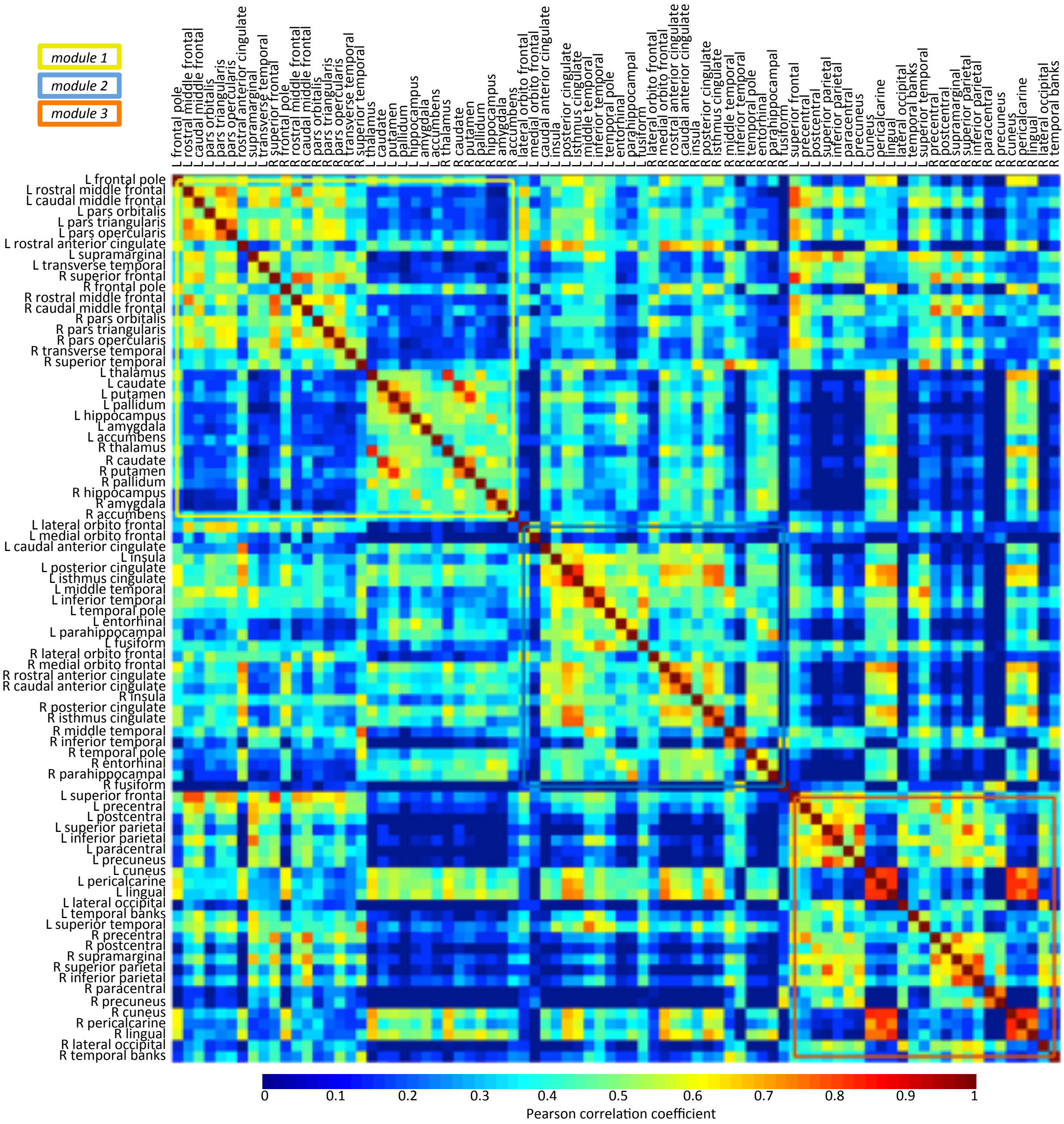


**Supplementary Figure 2. Weighted correlation matrix (Typical AD).** The brain regions were ordered by the brain modules identified in the healthy controls (Table 2 and Figure 3 in the main text). The yellow square (upper-left corner) indicates the brain regions belonging to module 1. The blue square (center) indicates the brain regions belonging to module 2. The orange square (bottom-right corner) indicates the brain regions belonging to module 3.

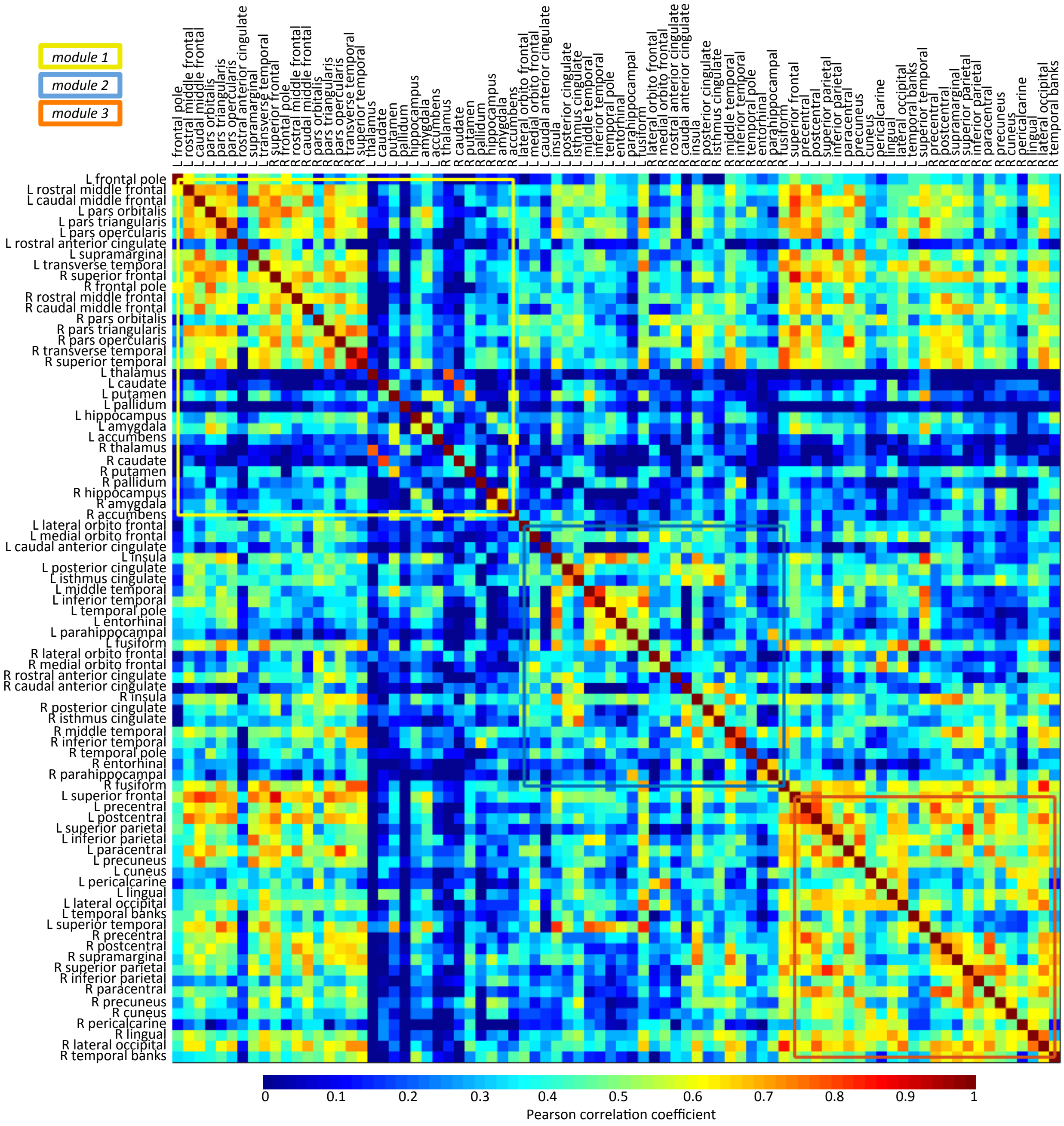


**Supplementary Figure 3. Weighted correlation matrix (Limbic-predominant AD).** The brain regions were ordered by the brain modules identified in the healthy controls (Table 2 and Figure 3 in the main text). The yellow square (upper-left corner) indicates the brain regions belonging to module 1. The blue square (center) indicates the brain regions belonging to module 2. The orange square (bottom-right corner) indicates the brain regions belonging to module 3.

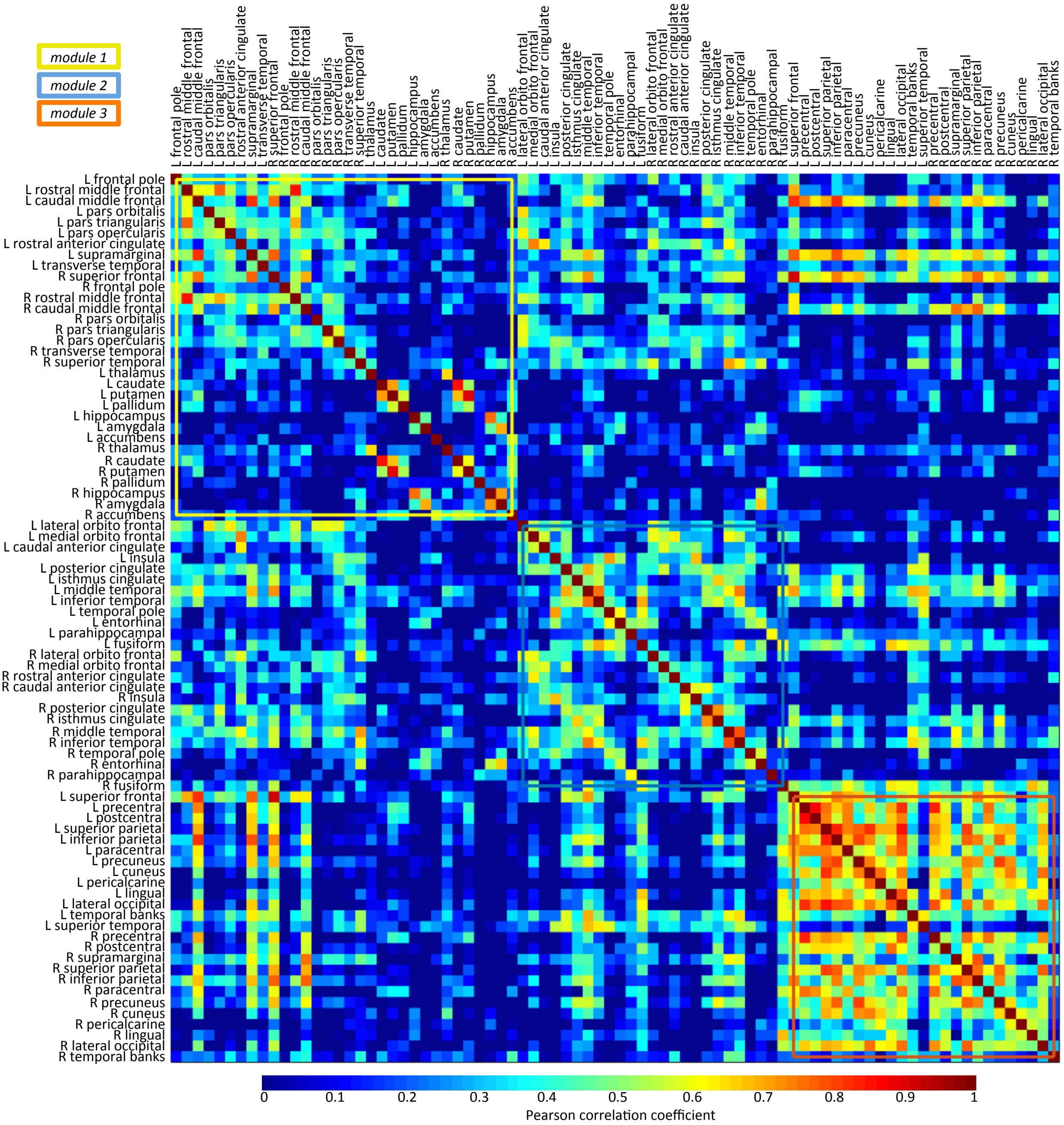


**Supplementary Figure 4. Weighted correlation matrix (Hippocampal-sparing AD).** The brain regions were ordered by the brain modules identified in the healthy controls (Table 2 and Figure 3 in the main text). The yellow square (upper-left corner) indicates the brain regions belonging to module 1. The blue square (center) indicates the brain regions belonging to module 2. The orange square (bottom-right corner) indicates the brain regions belonging to module 3.

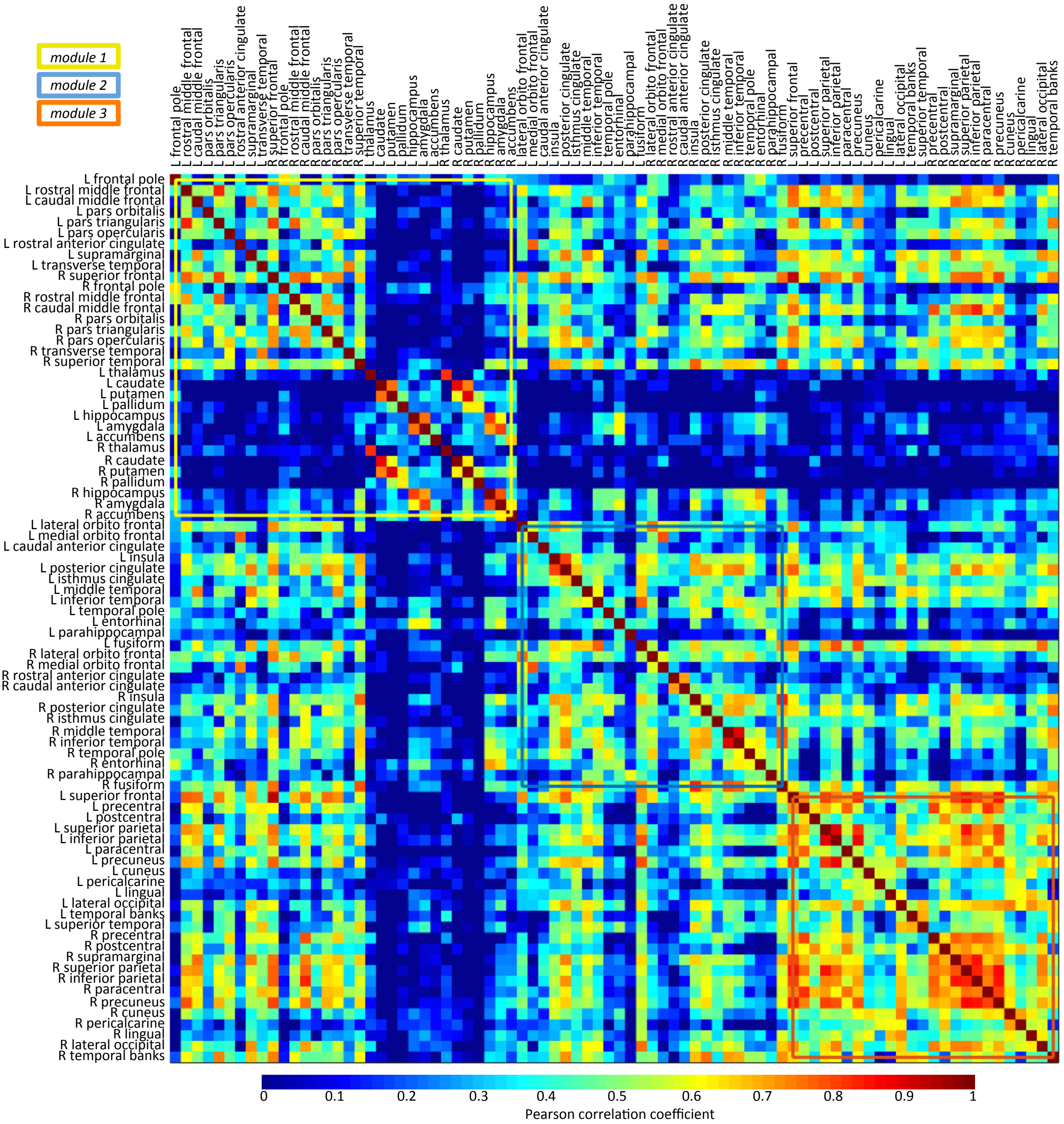


**Supplementary Figure 5. Weighted correlation matrix (Minimal atrophy AD).** The brain regions were ordered by the brain modules identified in the healthy controls (Table 2 and Figure 3 in the main text). The yellow square (upper-left corner) indicates the brain regions belonging to module 1. The blue square (center) indicates the brain regions belonging to module 2. The orange square (bottom-right corner) indicates the brain regions belonging to module 3.
